# Suicide in narcissism: Can shame-proneness make a difference?

**DOI:** 10.1192/j.eurpsy.2021.1563

**Published:** 2021-08-13

**Authors:** S. Beomonte Zobel, V. Mirizio, P. Velotti

**Affiliations:** 1 Dynamical And Clinical Psychology, Sapienza Università di Roma, Rome, Italy; 2 Prevention And Early Interventions In Mental Health, Asl Roma1, Rome, Italy

**Keywords:** Suicide, Suicide ideation, shame-proneness, Narcissism

## Abstract

**Introduction:**

Cluster B personality disorders are characterized by a higher prevalence of suicidal ideation and behavior than others, and Narcissistic Personality Disorder is no different. Very intense feelings of shame, intolerable for the individual, are often found in patients with Narcissistic Personality Disorder and may have a role in suicidal behavior.

**Objectives:**

To offer preliminary empirical evidences concerning the relationship between narcissism, shame and suicide ideation.

**Methods:**

We administered Pathological Narcissism Inventory (PNI), Test of Self Conscious Affects (TOSCA) and Beck Scale for Suicidal Ideation (BSI) to a sample of individuals with Suicide ideation (n= 65) and a sample of community participants (n=65).

**Results:**

Controlling for age and gender, in the merged sample we found that BSI scores correlated significantly with the vulnerable dimension of narcissism and with TOSCA Interpersonal Shame subscale. In the clinical sample, Interpersonal shame partially mediates the relationship between vulnerable narcissism and suicidal ideation.

**Conclusions:**

Shame seems to play a key role in the relationship between the vulnerable facet of narcissism and suicidal ideation, although the profound mechanism by which it works remains to be understood. Future directions and clinical implications are discussed.

